# Prevalence of Dyslipidaemia among Type 2 Diabetes Mellitus Patients in the Western Cape, South Africa

**DOI:** 10.3390/ijerph17238735

**Published:** 2020-11-24

**Authors:** Elizabeth I. Omodanisi, Yibanathi Tomose, Benjamin I. Okeleye, Seteno K. O. Ntwampe, Yapo G. Aboua

**Affiliations:** 1 Bioresource Engineering Research Group (BioERG), Faculty of Applied Sciences, Cape Peninsula University of Technology, P.O. Box 652, Cape Town 8000, South Africa; ben_okeleye2005@yahoo.com (B.I.O.); karabo.ntwampe@nwu.ac.za (S.K.O.N.); 2 Department of Biomedical Science, Faculty of Health and Wellness, Cape Peninsula University of Technology, Cape Town 7535, South Africa; yibanathit@gmail.com; 3 School of Chemical and Minerals Engineering, North-West University, Private Bag X1290, Potchefstroom 2520, South Africa; 4 Department of Health Sciences, Faculty of Health and Applied Sciences, Namibia University of Science and Technology, Windhoek 13388, Namibia

**Keywords:** diabetes mellitus, dyslipidaemia, cholesterol, high-density lipoproteins, low-density lipoproteins, triglyceride

## Abstract

Dyslipidaemia, an irregular aggregate of lipids in the blood is common in diabetes and cardiovascular disease sufferers. A cross-sectional study on the prevalence of dyslipidaemia was performed among type 2 diabetes mellitus (T2DM) patients in the Western Cape, South Africa. Patients (*n* = 100) that participated in the study were within the age range of 19–68 years, of whom 89% were observed to have serum lipid abnormalities. Out of the 100 patients, 56%, 64%, 61%, and 65% were recorded to have high total cholesterol (TC), hypertriglycemia, increased low-density lipoproteins cholesterol (LDL-C), and reduced high-density lipoproteins cholesterol (HDL-C), respectively. In male diabetic patients, a marked prevalence of (94%) dyslipidemia was noted, of which 52% were affected by high TC (5.3–7.9 mmol/L), with 70% having a high level of triglyceride (TG) [1.72–7.34 mmol/L], while 60% had a high LDL-C (3.1–5.5 mmol/L), including 78% with low HDL-C (0.7–1.1 mmol/L). In comparison, 84% of diabetic females had dyslipidemia, with high TC (5.1–8.1 mmol/L), hypertriglycemia (1.73–8.63 mmol/L), high LDL-C (3.1–5.6 mmol/L), and low levels of HDL-C (0.8–1.1 mmol/L) affecting 60%, 58%, 62%, and 52% of the patients, respectively. This study showed the importance of screening and the regular surveillance of dyslipidaemia in T2DM patients as there is a paucity of data on it in Africa.

## 1. Introduction

One of the major causes of death worldwide is cardiovascular disease (CVD), with an estimate of 17.9 million deaths in 2015 [1]. Diabetes is an increasing health challenge throughout the world, with up to 422 million people been diagnosed with diabetes worldwide in 2014 [2] and 80% of the population in developing countries being diagnosed with diabetes. According to the WHO, death caused by diabetes will double by the year 2030 [3]. Approximately, 285 million people worldwide were affected by diabetes in 2010, and by 2030, over 438 million people will be diagnosed with diabetes [4]. 

In South Africa, diabetes has been seen to be increasing. The Western Cape is the second diabetes dominant province compared to other provinces in South Africa [5,6]. The mixed-race population of South Africa was found to have the second-highest prevalence of diabetes after the Indian population [6]. Furthermore, some studies have shown abnormal lipid profiles in most diabetic cases. The abnormal metabolism of glucose, proteins, and lipids in diabetic patients is as a result of insulin deficiency or inadequate response in target [7,8]. This metabolic anomaly could be responsible for the abnormal lipid profile in diabetic patients. Different risk factors, such as physical inactivity, poor diet, smoking, and obesity can lead to Type 2 diabetes mellitus (T2DM). Several complications, such as oxidation stress, inflammation, and metabolic dysfunction, are associated with T2DM which eventually leads to cardiovascular risk in diabetic patients. Patients with diabetes have also been determined to have a high level of inflammatory cytokines [9,10].

The onset of diabetes involves several processes, varying from the autoimmune destruction of the pancreatic beta (β)-cells responsible for insulin production to abnormalities that cause insulin action resistance. Both insulin deficiency and resistances coexist in some cases of diabetes [9]. The majority of diagnosed diabetic cases fall under two categories: type 1 and type 2 diabetes mellitus [5]. Type 1 diabetes mellitus (T1DM), also known as insulin-dependent diabetes, is classified as a deficiency of insulin secretion. It counts for 5–10% of the different types of diabetes and is the most common in children and adolescence. Type 2 diabetes mellitus (T2DM), previously known as non-insulin dependent diabetes, is caused by the resistance of tissue to insulin action and an inadequate compensatory insulin secretory response. T2DM accounts for 90–95% of the type of diabetes observed in patients [7,8]. A different category of diabetes called gestational diabetes mellitus (GDM) is also insulin resistant but it occurs during pregnancy. In patients with GDM, diabetes usually self corrects without any treatment or intervention postnatally, but in certain cases, it can develop into T2DM [7]. The risk factors associated with GDM are obesity, a family history of GDM, or glycosuria. In certain cases of diabetes, there have been events of coronary heart disease (CHD), which are as a result of abnormal lipid profiles, known as dyslipidaemia [11]. 

A lipid profile is made up of three types of lipids, namely triglycerides (TG), cholesterol, and lipoproteins, which are all synthesised and excreted by the liver [5,12]. TG are types of lipids constituted by a glycerol backbone and different fatty acids, while cholesterol is an unsaturated steroid alcohol lipid present in human diet. Cholesterols and TG are transported by very-low-density lipoproteins (VLDL), low-density lipoproteins (LDL), high-density lipoproteins (HDL), and chylomicrons [13,14,15]. Chylomicrons are carriers of large quantities of exogenous TG and a minute quantity of cholesterol from the small intestines to the liver. Similarly, VLDL is responsible for carrying endogenous TG and cholesterol from the liver to body tissues. LDL carries cholesterol and minute quantities of TG from the liver to body tissues. HDL, also known as “the good cholesterol”, is responsible for the transportation of cholesterol from body tissues to the liver (reverse transport) for excretion [5,16]. The malfunctioning of these lipoproteins (carriers) has been attributed to the poor management of T2DM, with less than 50% of patients having a normal glucose level reported in developed countries and less than 10% having normal lipid, blood pressure, and glucose levels worldwide [8,17], which is in line with the data collected in South Africa revealing that 67% of patients have glycosylated haemoglobin (HbA1c) greater than 7% and that insulin therapy has a short-term positive effect in T2DM, but a longer effect in T1DM [8]. 

Diabetic dyslipidaemia can be treated when detected early. It can be improved by glycaemic (glucose) regulation, low free fatty acid and the overproduction of VLDL by the liver. It can also be alleviated by weight loss, exercise, smoking cessation, a healthy diet, and pharmacologic therapy [8,18,19]. These regulation plans for diabetic dyslipidaemia could reduce the risk of cardiovascular conditions in diabetic patients [20]. This study was aimed at investigating the prevalence of dyslipidaemia in diabetic patients in the Western Cape, South Africa, as well as to assess the lipid profile components in association with biometric data, including biochemical markers of the mixed population race determined to have a high prevalence of diabetes in the Western Cape, South Africa. Although dyslipidaemia is often common in DM, this disorder is underdiagnosed and undertreated in patients. The biochemical markers assessed were fasting plasma glucose (FPG), glycosylated haemoglobin (HbA1c), total cholesterol (TC), high-density lipoproteins cholesterol (HDL-C), low-density lipoproteins cholesterol (LDL-C) levels, and triglyceride (TG). 

## 2. Materials and Methods 

### 2.1. Study Design and Subjects 

This study was a prospective, randomized, cohort and cross-sectional investigation. The samples were collected from 100 diabetic patients—50 men and 50 women within the age range of 19–68 years. The small size of the sample collected was due to the short period of time (6 months) permitted to conduct the study. Diabetic patients who fasted for at least 8 hours were included in the study, while the lipid results from a patient who did not fast, pregnant women, and patients with chronic diseases/disorders were excluded. All patients included in this study were diagnosed with Type 2 diabetes for a minimum of one year. The study was carried out at Pathcare Park N1 Chemistry Reference Laboratories (PPNCRL) for a period of 6 months, with the samples being collected from patients at local medical clinics and hospitals in the Western Cape, South Africa. The specimens were collected by qualified phlebotomists and permission to conduct the study was granted by the PPNCRL in the Western Cape, South Africa. All the experimental procedures described in this study were approved by the Department of Biomedical Science Laboratory, Cape Peninsula University of Technology, (CPUT), Bellville, South Africa (CPUT/BMS-EC2014/22). 

### 2.2. Experimental Analysis 

#### 2.2.1. Blood Preparation 

Blood samples were collected from participants after 8 h of fasting and samples for glycosylated haemoglobin were collected into ethylenediaminetetraacetic acid (EDTA) tubes. The blood samples (not haemolysed or lipemic) were centrifuged at 3000 rpm for 15 min within 2 h of collection. Separated plasma and serum for the estimation of biochemical markers were stored at −20 °C. 

#### 2.2.2. Glucose Analysis

Plasma glucose levels were measured by the hexokinase method with an enzymatic UV test using the Beckman Coulter AU 5800 Clinical chemistry analyzers (Beckman Coulter Inc., Brea, CA, USA). The reference intervals for plasma (fasting) in adults were 4.1–5.9 mmol/L. The generally accepted cut-off levels for the diagnosis of diabetes are: random plasma glucose (RPG) ≥ 11.1 mmol/L, fasting plasma glucose (FPG) ≥7.0 mmol/L, and 2-h post-load glucose of ≥11.1 mmol/L during an oral glucose tolerance test (OGTT). The UV test was linear within a concentration range of 0.6–45.0 mmol/L for serum and plasma. 

#### 2.2.3. Lipid Profile 

The serum lipid profile was determined using the Beckman Coulter AU 5800 Clinical chemistry analyzers (Beckman Coulter Inc., Brea, CA, USA) with the total cholesterol (TC), triglyceride (TG), high-density lipoproteins cholesterol (HDL-C), and low-density lipoproteins cholesterol (LDL-C) levels being quantified. 

#### 2.2.4. HbA1c Analysis 

The HbA1c was measured according to sigma metrics using Variant Turbo II HbA1c kit and analyzer (Cam, Bio-Rad Laboratories, Hercules, CA, USA). Variant II uses the principle of high-performance liquid chromatography (HPLC) for the separation and determination of normal and abnormal haemoglobin (Bio-Rad Laboratories, 2014). 

### 2.3. Statistics

Statistical analysis was performed using the Prisms Graph pad 5.0 (Graph Pad software for Windows, San Diego, CA, USA) to calculate the Mean ± SD (standard deviation), while the impaired t-test was used to compare the difference between the control (non-diabetic group) and diabetic groups. A *p*-value < 0.05 was considered to be statistically significant. 

## 3. Results 

The parameters evaluated and measured in mmol/L were fasting plasma glucose and the fasting lipid profile for TG, TC, LDL-cholesterol, and HDL-cholesterol. HbAc1 analysis was recorded as a percentage and control level in mmol/L. Table 1 states the normal reference ranges for all parameters of dyslipidemia analyzed. 

### 3.1. Prevalence and Lipid Profile of Diabetic Patients 

The dyslipidaemia prevalence of 94% was observed in diabetic male patients, with 52% having high TC of 5.3–7.9 mmol/L, 70% having high levels of TG (1.72–7.34 mmol/L), 60% having high LDL-C (3.1–5.5 mmol/L), and 78% having low HDL-C (0.7–1.1 mmol/L), as reflected by the mean values in Table 2 and Table 3 when compared to Table 1. Meanwhile, the prevalence of dyslipidaemia was recorded to be 84% in diabetic female patients with a high level of TC (5.1–8.1 mmol/L), hypertriglycemia (1.73–8.63 mmol/L), and LDL-C (3.1–5.6 mmol/L) in 60%, 58%, and 62% of patients, respectively, whereas 52% had low HDL-C (0.8–1.1 mmol/L) concentration, as represented by the mean values in Table 3 and Table 4 when compared to Table 1. Overall, the prevalence of 89% of all the diabetic patients had dyslipidaemia, of whom 56% were observed to be having high TC (5.1–8.1 mmol/L); albeit, 64% were determined to have high hypertriglycemia (1.72–8.63 mmol/L), while 61% had high LDL-C (3.1–5.6 mmol/L) and 65% had low HDL-C (0.7–1.1 mmol/L). 

The findings indicate that the high levels of one or more of TC, TG, LDL-C, and low level of HDL-C that are common in T2DM patients could result in dyslipidaemia prevalence, of which 94% (male), 84% (female), and 89% (total) were observed to show in this study. The abnormal metabolism of lipids in diabetic patients is as a result of insulin deficiency and an inadequate response in target tissues with other risk factors and several processes during onset of diabetes, involving autoimmune destruction of the pancreatic beta (β)-cells and abnormalities that cause insulin resistance. 

### 3.2. Fasting Glucose Concentrations 

**Figure 1:**
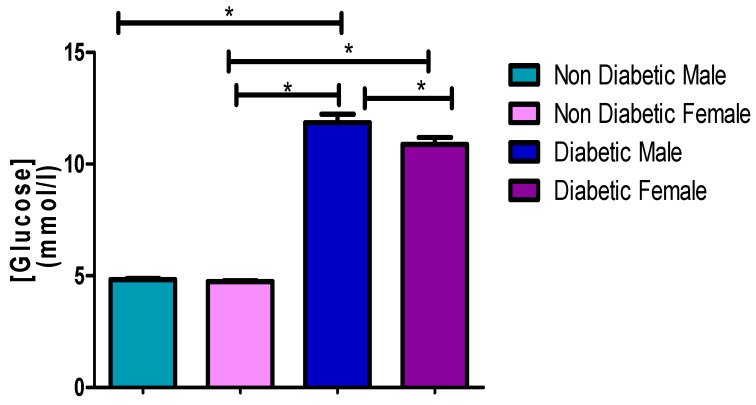
Fasting glucose concentrations. *, a significant statistical difference (*p*-value < 0.05). The statistical bar (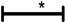) shows the relationship between the four variables: there was a significant statistical difference in the fasting glucose concentration between the non-diabetic male and diabetic male, the non-diabetic female and diabetic male, the non-diabetic female and diabetic female, and between the diabetic male and diabetic female, respectively.

This investigation reveals that the fasting glucose concentrations of the diabetic patients of the male (11.70 mmol/L) and female (10.88 mmol/L) patients that participated in this study were higher than the non-diabetic male (4.84 mmol/L) and female (4.74 mmol/L) groups with a very high significant difference (*p* ≤ 0.0001), respectively (Figure 1 and Table 2, Table 3 and Table 4). Moreover, the blood of diabetic males had a higher glucose concentration (11.70 mmol/L) when compared to diabetic females (10.88 mmol/L) with a slight significant (*p* = 0.0465) difference (Figure 1 and Table 3). 

### 3.3. Total Cholesterol Concentration

**Figure 2:**
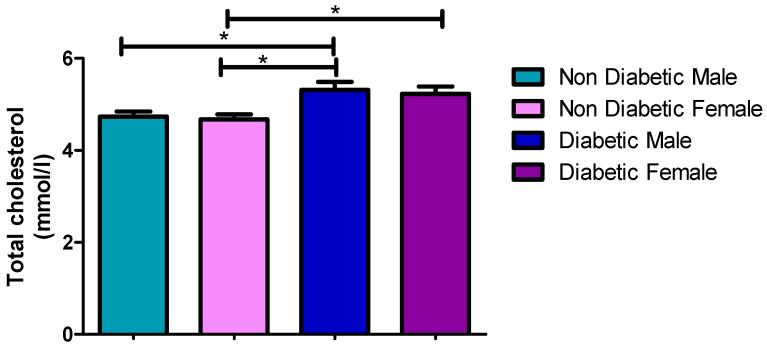
Total cholesterol concentration. *, a significant statistical difference (*p*-value < 0.05). The statistical bar (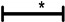) shows the relationship between the four variables: there was a significant statistical difference in the total cholesterol concentration between the non-diabetic female and diabetic female, the non-diabetic male and diabetic male, and between the non-diabetic female and diabetic male, respectively.

The TC concentration was higher in the diabetic patient with 5.23 mmol/L than in the non-diabetic patients of 4.67 mmol/L, with a statistical difference of *p* = 0.0086 (Figure 2, Table 2). Meanwhile, the level of TC was similar in both males (5.33 mmol/L) and females (5.23 mmol/L), with no statistical difference (*p* = 0.7159) (Table 3) between the patients. Furthermore, the TC levels were lower in non-diabetic males (4.73 mmol/L) than in diabetic (5.33 mmol/L) male patients (*p* = 0.0023), as shown in Table 2, with similar patterns being observed in female (*p* = 0.0086) patients (Table 3 and Table 4). 

### 3.4. Triglycerides Concentrations 

**Figure 3:**
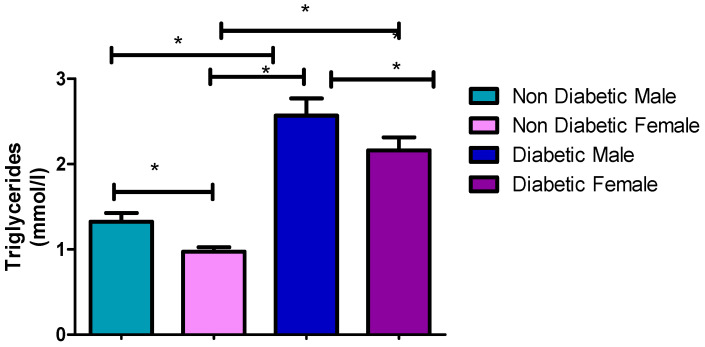
Triglycerides concentrations. *, a significant statistical difference (*p*-value < 0.05). The statistical bar (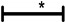) shows the relationship between the four variables: there was a significant statistical difference in the triglycerides concentrations between the non-diabetic female and diabetic female, the non-diabetic male and diabetic male, the non-diabetic female and diabetic male, the diabetic male and diabetic female, and between the non-diabetic male and non-diabetic female, respectively.

Observations (2.57 mmol/L) of TG were made in male diabetic patients who had a higher TG compared to non-diabetic males with a TG value of 1.32 mmol/L at a significant statistical difference of *p* = <0.0001. Similarly, in females, 1.39 mmol/L and 0.97 mmol/L were recorded for diabetic and non-diabetic patients, respectively (*p* = 0.0001). There was no significant difference (*p* = 0.3004) in the TG concentration of both male (2.57 mmol/L) and female (2.29 mmol/L) diabetic patients (Figure 3 and Table 2, Table 3 and Table 4). 

### 3.5. LDL-C Concentrations 

**Figure 4:**
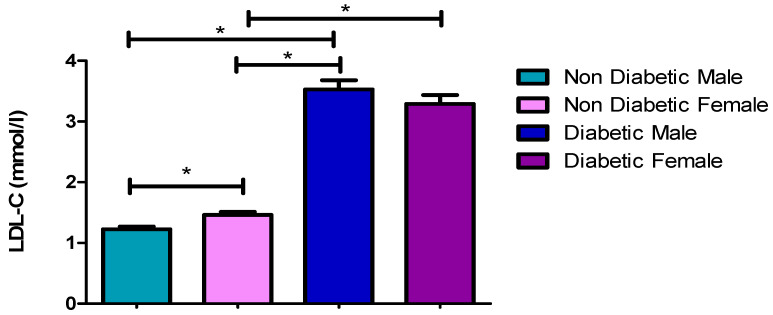
Low-density lipoproteins cholesterol (LDL-C) concentrations. *, a significant statistical difference (*p*-value < 0.05). The statistical bar (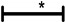) shows the relationship between the four variables: there was a significant statistical difference in the LDL-C concentrations between the non-diabetic female and diabetic female, the non-diabetic male and diabetic male, the non-diabetic female and diabetic male, and between the non-diabetic male and non-diabetic female, respectively.

Figure 4 and Table 2, Table 3 and Table 4 showed significantly higher LDL-C concentrations of diabetic male patients (3.53 mmol/L) and females (3.29 mmol/L) compared (*p* = 0.0082, *p* = 0.0120) to non-diabetic males (3.08 mmol/L) and females (2.84 mmol/L), respectively. Additionally, there were significantly higher LDL-C concentrations in diabetic males and females when compared to the non-diabetic females and males. However, no significant difference (*p* = 0.2293) was found when comparing the LDL-C concentrations between diabetic male (3.53 mmol/L) and female (3.29 mmol/L) patients. 

### 3.6. HDL-C Concentrations 

**Figure 5:**
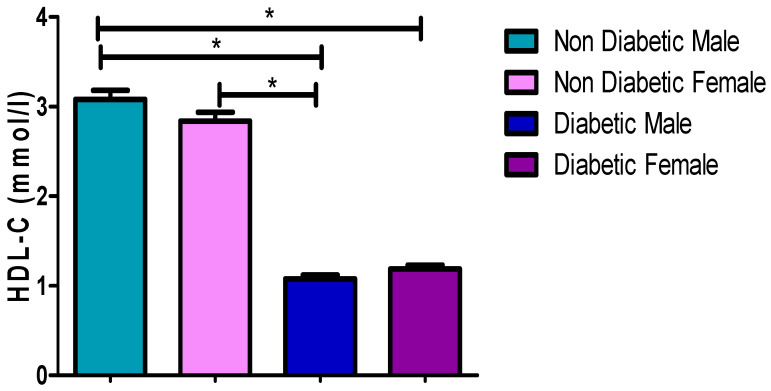
High-density lipoproteins cholesterol (HDL-C) concentrations. *, a significant statistical difference (*p*-value < 0.05). The statistical bar (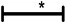) shows the relationship between the four variables: there was a significant statistical difference in the HDL-C concentrations between the non-diabetic male and diabetic female, the non-diabetic male and diabetic male, and between the non-diabetic female and diabetic male, respectively.

Figure 5 and Table 2, Table 3 and Table 4 showed that HDL-C concentration in diabetic male patients (1.08 mmol/L) and females (1.21 mmol/L) was significantly (*p* = 0.0135, *p* = 0.0004) lower than those observed for non-diabetic males (1.23 mmol/L) and females (1.46 mmol/L), respectively. However, a significant difference was found when comparing the HDL-C concentrations between diabetic males (1.08 mmol/L) and females (1.21 mmol/L), whereby the female had a higher HDL-C compared (*p* = 0.0442) to males. 

### 3.7. Total Chol/HDL-C Concentrations 

**Figure 6:**
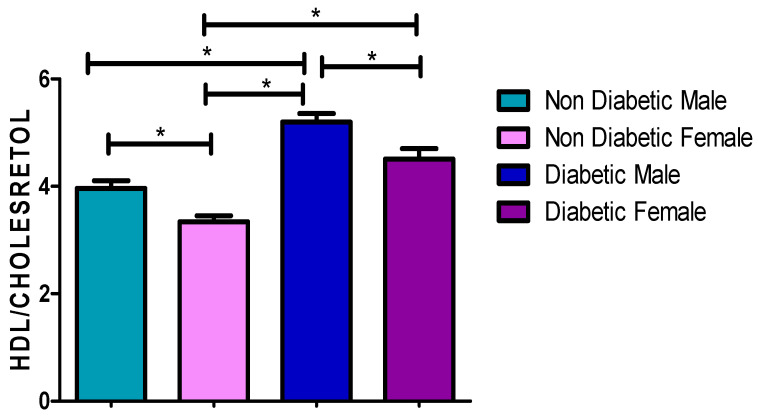
Cholesterol/HDL-C ratio. *, a significant statistical difference (*p*-value < 0.05). The statistical bar (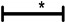) shows the relationship between the four variables: there was a significant statistical difference in the Cholesterol/HDL-C ratio between the non-diabetic female and diabetic female, the non-diabetic male and diabetic male, the diabetic male and diabetic female, the non-diabetic female and diabetic male, and between the non-diabetic male and non-diabetic female, respectively.

Figure 6 and Table 2, Table 3 and Table 4 showed a significantly higher Chol/HDL-C ratio in diabetic males (5.13 mmol/L) and females (4.68 mmol/L) compared to non-diabetic male (3.96 mmol/L) and female (3.34 mmol/L) patients (*p* ≤ 0.0001), respectively. In addition, there was a significantly higher Chol/HDL-C ratio in diabetic males and females compared to non-diabetic females and males. However, no significant difference was found when comparing the Chol/HDL-C ratio between diabetic male (5.13 mmol/L) and female (4.68 mmol/L) patients (*p* = 0.1042). 

## 4. Discussion 

T2DM may trigger disorders whereby the majority of the lipid profile results are abnormal; for example, 89% of the patients had dyslipidaemia in this study. This shows that dyslipidaemia was prevalent in most of the diabetic patients. The most affected lipid profile component in DM is HDL, as the lack of insulin action increases triglyceride-rich molecules, which increase the exchange of the HDL to LDL and accelerate its excretion, as evidenced by the 78% of male and 62% of female T2DM patients who had low HDL. The entire group of T2DM patients had poor control of the diabetic disorder, as revealed by an HbA1c greater than 6.1% in all of the diabetic patients participating in the study when compared to non-diabetic participants, i.e., with averages of 9.4% and 9.0% for male and females, albeit with minimal differentiation between the two groups. This study recorded the prevalence of 89% of dyslipidaemia among T2DM in Cape Town, South Africa, a region with a mixed-race population (Black, White, and Mixed ancestries) and genetic heterogeneity, such as in the Mixed ancestries population group whose ancestry is about 9–11% Asian, 20–36% Black, 32–43% Khoisan, and 21–28% White [6]. However, the prevalence (89%) is in line with a study conducted in Durban, South Africa, with over 90% of the black patients with T2DM showing one or more lipid abnormalities of dyslipidaemia (cholesterol and triglyceride) [6,22]. Meanwhile higher prevalence of dyslipidaemia (93.5%) was observed among T2DM patients in a study conducted in the Johannesburg area of South Africa [23]. 

This study was comparable to other studies in African diabetic patients, with similar results being reported in a study at Kenyatta National Hospital, Nairobi, which showed that over 70% of the participants had a total cholesterol level greater than 4.2 mmol/L, LDL greater than 2.6 mmol/L, and an HDL level lower than 1.00 mmol/L. Only a few (28.3% males and 32.2% females) of the participants had a triglyceride level greater than 1.7 mmol/L [11,23,24]. In this study, a prevalence of 89% of dyslipidaemia in all the T2DM patients, 56% with high cholesterol of 5.1–8.1 mmol/L, was observed, with 64% having hypertriglycemia of 1.72–8.63 mmol/L, while 61% were affected by a high LDL cholesterol of 3.1–5.6 mmol/L. Only 65% of the patients had a low HDL of 0.7–1.1 mmol/L. 

A study conducted in Nigeria with 600 patients revealed 89% dyslipidaemia in the population studied, which was a similar result to that being reported herein, albeit with a lower population (*n* = 100) of diabetic patients [25]. The Nigerian study results included an elevated LDL (74%), total cholesterol (43%), 13% of TG, and a 53% decrease in HDL. These studies were similar to a study conducted in India whereby a high total cholesterol in diabetic patients with dyslipidaemia prevelance of 86% in patients was observed. The HDL dyslipidaemia in the Indian study was found to be 71%, with LDL dyslipidaemia being observed in 64% of the diabetic patients, while hyper- tryglyceridemia was found in 47% of the participants [25,26]. Similarly, in a study in Botswana whereby 401 diabetic patients were involved, 33.5% had high TC and 38.9% had high TG, with 41% and 47% suffering from hypercholesterolemia and hypertriglyceridemia, respectively [27], which is lower in prevalence when compared to the present study, as reported herein, whereby a high prevalence of TC (56%), hypertriglycemia (64%), LDL-C (61%) and a low HDL-C (65%). As many other researchers reported similar results, this is evidence that T2DM patients have a significant complication of dyslipidaemia, which, as a result, may culminate in coronary artery diseases or cardiovascular disease (CAD/CVD), which may result in an even higher mortality rate among these patients. 

Commonly observed abnormalities that occurred together in diabetic patients were also documented by other studies, specifically elevated TG and low HDL-C [25,26]. This was corroborated with the findings observed in this study, with 49% of the patients having both elevated TG and a low HDL-C. This is regarded as dyslipidaemia-associated T2DM, which clearly proves that high levels of TG have a direct effect in lowering the HDL-C levels in the body. Sixty-nine percent of the diabetic patients in this study had a cholesterol/HDL (Chol/HDL) ratio greater than 4 (mean 4.906). Eighty percent (40/50) of the males had a mean Chol/HDL ratio of 5.128 (Figure 6) with fifty-eight percent (29/50) of the females having a mean Chol/HDL ratio of 4.684 (Figure 6 and Table 2, Table 3 and Table 4). It is believed that a Chol/HDL ratio greater than 4 is a possible pre-atherogenic state; therefore, most of these patients have a greater chance of developing atherosclerosis leading to CAD/CVD. 

Figure 1, Figure 2, Figure 3, Figure 4 and Figure 5 clearly show that T2DM males had a higher concentration of glucose, TC, TG, LDL-C and Cholesterol/HDL ratio, respectively, compared to the non-diabetic group. These values were also slightly higher than those observed for diabetic females. Meanwhile, the male T2DM group showed a lower concentration of HDL-C compared to the non-diabetic group, with the concentration values being slightly lower than T2DM females, as presented in Figure 6. Figure 1, Figure 2, Figure 3, Figure 4 and Figure 5 also reveal that diabetic females have a significantly higher concentration of fasting glucose, TC, LDL-C, and chol/HDL compared to the non-diabetic group, which was also slightly lower than T2DM males. It has been reported that females are more prone to diabetic dyslipidemia which is atherogenic than that of ordinary dyslipidemia as compared to males and hence have more risk of developing atherosclerosis with increasing age [28]. 

The comparison made between diabetic male and females in the previous study found that most of the parameters were statistically insignificant, with *p*-values of greater than 0.05 [24]. This is in line with this study whereby there were several insignificant statistical differences between diabetic males and females in some instances (Table 3), with the highest in TC. The *p*-values for TC (*p* = 0.7159), TG (*p* = 0.3004), LDL-C (*p* = 0.2293), and Chol/HDL ratio (*p* = 0.1042) were all statically insignificant. Glucose (*p* = 0.0465) and HDL (*p* = 0.0442) were the only two parameters that were found to be statistically significant. This meant that T2DM males have had higher levels of glucose and lower level of HDL-C compared to females, but with no significant difference in other parameters. Moreover, high glucose and low HDL-C are significant occurrences in T2DM patients, and those with the high blood TG usually also have lower level of HDL cholesterol compared to other parameters in both males and females, which contributes to the worsening of diabetic (glycemic) control and increased cardiovascular risk associated with this condition; hence, this constitutes a twofold reason for controlling the level of HDL-C in patients with T2DM [29,30]. 

## 5. Conclusions 

A large number of the participants in this study showed rudimentary glycemic control and had dyslipidaemia with an increase in total cholesterol, triglyceride, and LDL, with a noticeable decrease in HDL when compared to non-diabetic participants. The results in this study have clearly corroborated the results of previous studies that diabetic patients have a serious complication of dyslipidaemia. However, these results may not be a full representation of all South Africans but it is necessary to note that similar studies also reported related results in other African countries. We hope that the data in this study will help to demonstrate the importance of regular surveillance and screening of all diabetic patients for dyslipidaemia. 

## Figures and Tables

**Table 1:** Normal reference ranges for biomarkers of dyslipidemia [21].

Parameters	Ranges
Fasting glucose	3.5–5.5 mmol/L
HbA1c	3.9–6.1%
Triglycerides	<1.7 mmol/L
Total cholesterol	<5.0 mmol/L
LDL- cholesterol	<3.0 mmol/L
HDL- cholesterol	>1.2 mmol/L

**Table 2:** Non-diabetic and diabetic male patient’s biomarkers of dyslipidemia.

Parameters	Non-Diabetic		Diabetic		*p*-Value
	Mean (mmol/L)	SD *	Mean (mmol/L)	SD	
Fasting glucose	4.84	0.28	11.70	2.56	<0.0001
Total cholesterol	4.73	0.75	5.33	1.23	0.0023
Triglyceride	1.32	0.72	2.57	1.42	<0.0001
LDL- chol	3.08	0.71	3.53	1.04	0.0082
HDL-chol	1.23	0.32	1.08	0.31	0.0135
Chol/HDL ratio	3.96	1.01	5.13	1.21	<0.0001
HbA1c	ND *	ND	9.38	0.21	ND

* SD, standard deviation; ND, not determined.

**Table 3:** Diabetic male and female patients.

Parameters	Diabetic Male		Diabetic Female		*p*-Value
	Mean (mmol/L)	SD *	Mean (mmol/L)	SD	
Fasting glucose	11.70	2.56	10.88	2.20	0.0465
Total cholesterol	5.33	1.23	5.23	1.14	0.7159
triglyceride	2.57	1.42	2.29	1.39	0.3004
LDL- chol	3.53	1.04	3.29	1.04	0.2293
HDL-chol	1.08	0.31	1.21	0.32	0.0442
Chol/HDL ratio	5.13	1.21	4.68	1.50	0.1042
HbA1c	9.38	0.21	8.97	1.75	0.2381

* SD, standard deviation; ND, not determined.

**Table 4:** Non-diabetic and diabetic female patient’s biomarkers of dyslipidemia.

Parameters	Non-Diabetic		Diabetic		*p*-Value
	Mean (mmol/L)	SD *	Mean (mmol/L)	SD	
Fasting glucose	4.74	0.31	10.88	2.20	<0.0001
Total chol	4.67	0.77	5.23	1.14	0.0086
triglyceride	0.97	0.37	2.30	1.39	<0.0001
LDL-chol	2.84	0.71	3.29	1.04	0.0120
HDL-chol	1.46	0.36	1.21	0.32	0.0004
Chol/HDL ratio	3.34	0.77	4.68	1.50	<0.0001
HbA1c	ND *	ND	8.97	1.75	ND

* SD, standard deviation; ND, not determined.
